# Making data count

**DOI:** 10.1038/sdata.2015.39

**Published:** 2015-08-04

**Authors:** John E. Kratz, Carly Strasser

**Affiliations:** 1 California Digital Library, 415 20th Street, 4th Floor Oakland, CA 94612-2901, USA; 2 Gordon and Betty Moore Foundation, 1661 Page Mill Road, Palo Alto, CA 94304, USA

**Keywords:** Databases, Data publication and archiving

## Comment

Science is built on a foundation of data. The production and publication of that data should be recognized as valuable scholarship, but data lacks an essential prerequisite for modern-day scholarly recognition—accepted metrics of significance. The scientific community has traditionally estimated the impact of a journal article by counting the number of subsequent references to it; more recently, a suite of web-based alternative metrics (‘altmetrics’) offer faster assessment and the chance capture other kinds of impact^1^. Data can be fit into these article-centered assessment systems by proxy, via data descriptor articles in journals like *Earth Systems Science Data* or *Scientific Data*^2,3^. Another approach is to apply familiar metrics directly to datasets published in online databases or repositories. Complicating matters, the same metric may mean different things with respect to articles versus datasets. A researcher can read an article online closely without downloading the PDF version, but if they view a dataset landing page without downloading the data, their level of engagement is almost certainly low. A better understanding of how to measure data impact is critical if we are to reward data creators and incentivize data publication.

In recognition of this need, several groups are currently investigating traditional, alternative, and novel metrics for data. The joint Research Data Alliance/World Data System Data Publishing Bibliometrics Working Group aims to ‘conceptualize data metrics and corresponding services’ (http://go.nature.com/BszO9f) by investigating current and potential applications for data metrics. The National Information Standards Organization (NISO) Alternative Assessment Metrics Initiative is working to define standards and best practices for applying altmetrics to non-traditional products like software and data (http://www.niso.org/topics/tl/altmetrics_initiative/). The Consortia Advancing Standards in Research Administration Information (CASRAI) Dataset Level Metrics Subject Group exists to facilitate communication and support interoperability among the various initiatives and stakeholders (http://casrai.org/standards/subject-groups/dataset-level-metrics). Here, we present research conducted for Making Data Count (MDC, http://mdc.lagotto.io/), a collaboration between the California Digital Library (CDL), the Public Library of Science (PLOS), and Data Observation Network for Earth (DataONE) to not only define, but implement a practical suite of data metrics.

## Survey and Demographics

To develop useful metrics, we must understand the needs and values of both the researchers who create and use data, and of the data managers who preserve and publish it. Before starting work on Making Data Count, we had conducted a survey of researcher perspectives on data publication that touched briefly on metrics of impact^4^. Respondents to that survey found citation and download counts much more useful than search rank or altmetrics. For this project, we expanded on that work with new surveys for researchers and data managers about data sharing, discovery, and metrics.

In November and December of 2014, we solicited responses to a pair of online surveys via social media, listservs, and posts to CDL and PLOS blogs—ultimately hearing from 247 researchers and 73 data managers.

- Data managers represented primarily academic (64%) and government-run (22%) repositories—in the United States (72%) or United Kingdom (11%).
- Most (78%) of the researchers were academics—working in the United States (57%) or United Kingdom (14%).
- Researchers covered the academic career spectrum: from principal investigators (42%), to postdocs (21%), and graduate students (19%).
- More than half (53%) were biologists, but environmental (17%) and social (10%) science were also well represented.

The survey was approved by the University of California, Berkeley Committee for Protection of Human Subjects/Office for the Protection of Human Subjects (protocol ID 2014-10-6794). Anonymized individual responses are available in the University of California's Merritt repository^5^.

## Sharing, Discovery, and Use

**Figure 1:**
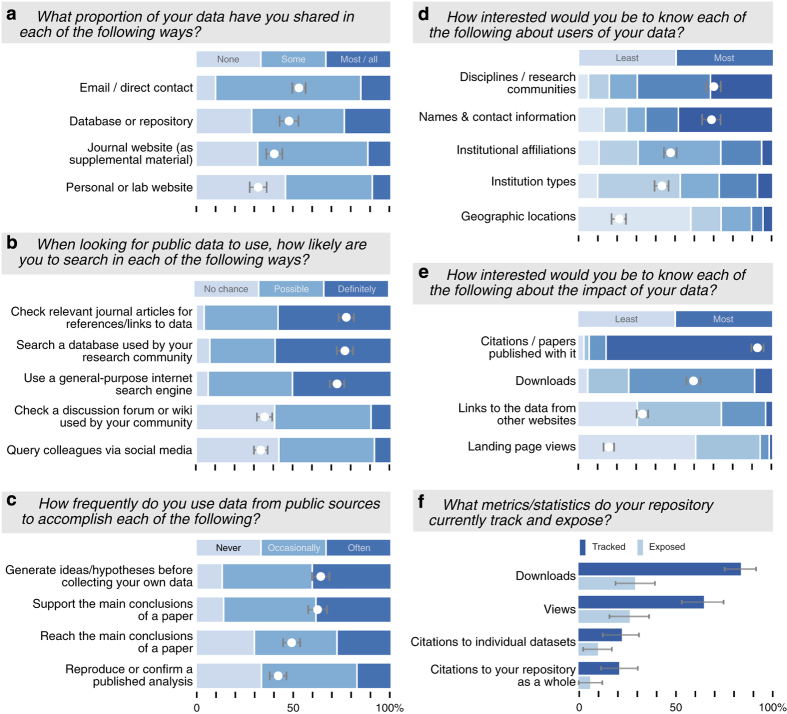
Researchers value citations and download counts; most repositories track downloads but few expose them. Researchers (*n*=247) indicated how they (**a**) share data, (**b**) search for data, and (**c**) subsequently use it. With respect to their own data, we asked what they would be most interested to know about (**d**) the users of the data and (**e**) the impact of that use. Finally, (**f**) data managers (*n*=71) reported which of these metrics their repositories track and expose. White dots show the mean on a scale of one-to-three (**a**,**b**,**c**), one-to-four (**e**), or one-to-five (**d**). All error bars depict 95% confidence intervals calculated by basic bootstrap with 10,000 resamplings.

Before we can collect data metrics, we need to know where to look. How do researchers share their data? How do they search for data to use? The question of data sharing behavior has been studied extensively^4,6–9^. As in previous studies, our respondents most often shared data directly in response to personal requests (Fig. 1a). Among the drawbacks to this practice—including the potential for capricious denial of access—is that it generally takes place over private channels (e.g., email) invisible to metrics. Fortunately, researchers do also often share over a visible channel: nearly 75% had published at least some data in a database or repository. Furthermore, although respondents used email more for sharing *some* data, repositories were more popular for sharing most or all of their data.

In contrast to sharing, where and how researchers search for data to (re-)use is relatively unknown. To investigate, we asked researchers how likely they would be to use each of five possible search strategies for publicly accessible data (Fig. 1b). Rather than relying on any single method, most researchers (63%) would ‘definitely’ employ multiple strategies. Three predominated: checking the literature for references, searching a discipline-specific database, or a using general purpose search engine (e.g., Google). Given the opportunity to write-in particular sources, respondents most frequently (*n*=16) mentioned Dryad; Google and ‘journal articles’ tied for second (*n*=14). Researchers generally did not `crowd-source' data discovery via inquiries on social media or discussion forums. Direct inquiries (by email or in person) to knowledgeable colleagues were a frequent (*n*=12) write-in source and are probably quite common, although (like sharing via email) invisible to metrics.

After scientists find public data, how do they use it? We asked researchers how frequently they used public data at the outset (to generate ideas/hypotheses), as the central piece (to reach a main conclusion), or towards the end (to support a main conclusion) of their research process (Fig. 1c). An overwhelming 96% of respondents at least ‘occasionally’ use public data at some point in the process, and a majority used public data ‘often.’ Although use in one of the supporting roles was more common, a 70% majority at least occasionally use public data to reach main conclusions. Researchers consider the centrality of a dataset to their own work in deciding how to credit the creators^4^, so these different uses are likely to generate different indicators of impact. We saw use of published data for a variety of purposes by virtually all respondents, highlighting the critical role of public data in how science is done today.

## (re)Users

Researchers who share data want to know who is using their data for what purpose^6,10^, and this understandable wish may contribute to the popularity of sharing on request. Public repositories could at least partially satisfy depositor interest while still making data openly available by collecting some amount of information about data users. When we asked researchers what they want to know about users of their data, nearly half selected the most detailed option, ‘name and contact information,’ as their first choice (Fig. 1d). Researchers expressed little interest in communities defined by geography or affiliation, but average interest in knowing a user's scientific discipline was just as high as in knowing their name. Discipline was even more clearly a priority for data managers; 67% of them ranked it first.

For practical purposes, we wanted to know how these preferences compare to current repository practices, so we asked data managers what information their repositories collect. We found that collection of user information is highly polarized. Half of the repositories require users to supply detailed contact information—names (47%) and email addresses (44%)—and half (47%) do not collect any information at all. On one hand, in cases involving sensitive data, repositories may need know users identities to verify that their requests are legitimate and to hold them to data use agreements; on the other, open and friction-less access to data makes it easier to use. As a compromise repositories might ask users about the discipline in which they plan to use a dataset. This would preserve user anonymity while providing data managers and creators with a valuable sense of the data is being used.

## Metrics of Impact

Although there are many potential audiences for data metrics, including administrators, funders, and data managers^11^, the most invested are undoubtedly the researchers who create them. Even if no information about data users is collected, metrics of use can demonstrate to researchers that their data is of interest and that the effort to share it was not wasted. Data managers also have a stake in knowing about use of their data to tailor services and justify funding. To learn about the immediate interests of these groups, we asked them to rank several potential metrics of impact (Fig. 1e).

Majorities of both researchers and data managers ranked landing page views as the least interesting metric. Researchers consistently ranked downloads as the second most interesting, while data managers (who were given a longer list to rank) put them in the middle. Resoundingly, both researchers and data managers measure scholarly prestige in citations; 85% of researchers and 61% of data managers ranked citations as the most interesting metric. This ordering—viewing, downloading, citing—is consistent with the results our previous researcher survey^4^. It depicts a ladder of increasing engagement with the data over time and suggests an entirely sensible weighting of metrics.

We asked data managers what metrics or statistics their repositories already track (Fig. 1f). A majority track landing page views and almost all track downloads. Despite high interest in citations, relatively few repositories track them, presumably because they are much more challenging to capture. Surprisingly few repositories report the metrics they track. Approximately one-third of the repositories that track each metric expose it through a programming interface or display it to site visitors. Limiting metrics to internal use shuts out many potential audiences and frustrates any cross-repository comparison of impact.

## Discussion

Citations are the coin of the academic realm, but their present usefulness for data is limited because datasets are rarely cited formally^12^. A 2011 survey of social science papers best illuminates current practice: only 17% of papers that used published data cited it in the reference list, roughly the same percentage as in 1995 (refs ^13,14^). However, we do believe that the situation will improve.

Researchers themselves strongly favor formal data citation. In a cross-disciplinary 2011 survey, 95% of respondents agreed that formal citation is a fair condition for data sharing, as did 87% of astrobiologists in a follow-up survey^6,9^. Citation ‘in the references like normal publications’ is the preferred method of receiving credit for data sharing by 71% of biodiversity researchers and by 75% of respondents to our earlier survey^4,15^.

In 2014, the scholarly communication community arrived at a Joint Declaration of Data Citation Principles with formal citation at its core^16^. The Joint Declaration has since been endorsed by 94 repositories, publishers, and scholarly organizations—including DataCite, CODATA, and Nature Publishing Group (http://go.nature.com/NHOqUp). *Scientific Data* data descriptors, for example, are formatted in accordance with the Joint Declaration, and each includes at least one formal data citation.

In contrast to citations, repositories can easily track data landing page views and downloads today. Neither researchers nor data managers put any weight on page views but, for researchers, downloads are a highly regarded second-choice metric. Responses to our previous survey suggest that the gap in perceived value between citations and downloads is surprisingly narrow^4^. Most repositories already track downloads, and we strongly recommend that more of them make download counts public.

## Conclusions

This survey provides several clear points of guidance for Making Data Count and other data metrics initiatives. In the short-term, page views and social media activity can be de-emphasized because of low status and lack of data-related activity respectively. While challenging, citations should be emphasized and collected as best as possible. Downloads should be emphasized as, at present, a happy medium: both reasonably valuable and reasonably easy to measure.

In response to background research—including the results we have presented here—we are adapting the existing PLOS Article Level Metric tool (http://alm.plos.org) to capture and present metrics for datasets. This tool currently collects data from 13 sources, including bookmarking services (e.g., CiteULike, Mendeley), social media (e.g., Facebook, Twitter), and DataCite metadata (http://mds.datacite.org/). One previously successful approach to capturing informal citations^17^ that we are taking in Making Data Count is to search the full text of articles in several open access corpora (BioMed Central, Europe PubMed Central, and PLOS) for dataset identifiers. We are also using the Nature OpenSearch API as an additional source of dataset citations in the scholarly literature.

As of this writing, we have imported 94,752 DataONE datasets; new datasets are being added as they are published. Modifications to the DataONE network now enable it to track COUNTER (http://www.projectcounter.org/) compliant download statistics and expose them via API for the DLM tool to collect. In the future, we plan to also import datasets published by the Dryad general-purpose repository (http://datadryad.org/). Once these steps are completed and the tool is able to compile the full range of metrics discussed here, the final phase of development—to be completed in the Fall of 2015—will be to present the outcome via a web-friendly reporting and visualization tool that gives users easy access to the data for further analysis.

While we would be pleased to see more sophisticated schemes to apportion scholarly credit and facilitate knowledge discovery^18–20^ catch on, these straightforward metrics fulfill an immediate need to quantify data impact in a way that all of the stakeholders—including data managers, administrators, and researchers—can understand today.

## Additional Information

**How to cite this article:** Kratz, J. & Strasser, C. Making data count. *Sci. Data* 2:150039 doi: 10.1038/sdata.2015.39 (2015).
